# Intimate partner violence during pregnancy against 601,534 women aged 15 to 49 years in 57 LMICs: prevalence, disparities, trends and associated factors using Demographic and Health Survey data

**DOI:** 10.1016/j.eclinm.2025.103382

**Published:** 2025-07-24

**Authors:** David Jean Simon, Vénunyé Claude Kondo Tokpovi, Adama Ouedraogo, Kassoum Dianou, Ann Kiragu, Comfort Z. Olorunsaiye, Bénédique Paul

**Affiliations:** a Centre de Recherches Appliquées et Interdisciplinaires sur les Violences Intimes, Familiales et Structurelles (RAIV), Laval University, Québec, Canada; b Groupe de Recherche sur l’inadaptation Psychosociale (GRIP), Laval University, Canada; c Laboratoire Printemps, University of Versailles - Saint-Quentin-en-Yvelines, Guyancourt, France; d University of Louvain, Louvain-La-Neuve, Belgium; e Department of Law and Political and Social Sciences, University of Sorbonne Paris Nord, Paris, France; f Department of Public Health, Arcadia University, Glenside, PA, USA; g AgroUniQ Lab, Université Quisqueya, Port-au-Prince, Haiti

**Keywords:** Intimate partner violence, Pregnancy, Disparities, Low- and middle-income countries

## Abstract

**Background:**

Pregnant women are at increased risk of intimate partner violence (IPV), with harmful outcomes for both mother and unborn baby. As this public health issue remained poorly documented in low- and middle-income countries (LMICs), this comprehensive study aimed to investigate prevalence and disparities of intimate partner violence during pregnancy (IPVDP) in 57 LMICs, grouped into WHO Regions and World Bank 2022 Income Classification. We also examined changes in IPVDP in LMICs and associated factors.

**Methods:**

Data for this study were extracted from Demographic and Health Surveys conducted in 57 LMICs from 2000 to 2024. Countries without domestic violence data were excluded from the study. We estimated overall, regional, sub-regional, and national-weighted prevalence of IPVDP among women aged 15–49 years. Trends in IPVDP were calculated at the national level using the average annual rate of change (AARC) in a subset of 31 countries with at least two survey rounds. A Poisson regression model was fitted for identifying factors associated with IPVDP.

**Findings:**

The study included a total weighted sample of 601,534 women. The pooled prevalence of IPVDP was 6.3% (95% CI 6.2–6.4). The Eastern Mediterranean region recorded the highest prevalence of IPVDP (11.3% [95% CI 10.8–11.9]), while the South-East Asia region (3.3% [95% CI 3.0–3.5]) the lowest. Similarly, low-income countries (LICs) reported the highest prevalence of IPVDP (8.2% [95% CI 7.9–8.4]). Further, prevalence varied greatly across countries, ranging from 1.1% (95% CI 0.6–1.5) in South Africa to 17.6% (95% CI 15.3–20.0) in Papua New Guinea. Examining AARC, most countries (23/31) experienced a decreasing trend in IPVDP, however, seven countries showed increasing trends, with the largest of 8.3% (95% CI 6.8–9.9) in Gambia and the smallest of 0.6% (95% CI –0.7 to 1.8) in the Democratic Republic of Congo. Similarly, we found that women: aged 15–19 years, from poor households, with primary education and below, who had more than 5 children, married before 18, who had not participated in household decision-making, and whose partners exhibited controlling behaviour had higher likelihood of experiencing IPVDP.

**Interpretation:**

Despite the implementation of numerous development programs aimed at reducing violence against women in LMICs in recent decades, IPVDP remains high in some countries and has even increased in others. Our findings indicate that efforts to reduce IPVDP and the associated health burdens need to be improved in many LMICs. In alignment with the 5.2 Sustainable Development Goal target (i.e., eliminate all forms of violence against women and girls), stakeholders such as NGOs and policymakers can use these findings to roll out interventions based on observed geographic and socio-demographic inequities to end IPVDP among vulnerable groups.

**Funding:**

The 10.13039/501100000156Fonds de Recherche du Québec-Santé (2022–2023—BF15—314279).

**Research in context**

**Evidence before this study**

On January 25, 2025, we searched PubMed for studies published that documented intimate partner violence during pregnancy (IPVDP) with no language restrictions. We used key and MeSH terms (“intimate partner violence”, OR “IPV”, OR “domestic violence”, OR “Gender-based violence”, OR “sexual violence”, OR “emotional violence”, OR “physical violence”, OR “intimate partner violence during pregnancy”, OR “sexual violence during pregnancy”, OR “emotional violence during pregnancy”, OR “physical violence during pregnancy”, OR “perinatal violence”) AND (“developing countries”, OR “low- and middle-income countries”, OR “LICs”, OR “MICs”, OR “LMICs”) to locate the studies. We found that the literature is dominated by studies conducted in developed countries.

**Added value of this study**

To our knowledge, this is the largest multi-country study documenting IPVDP utilizing nationally representative data from 57 low- and middle-income countries (LMICs). Moreover, it provides the trend analysis of IPVDP across countries. Geographical variations across LMICs were also noted.

**Implications of all the available evidence**

Our multi-country study showed that further concerted efforts are required across all governments and their partners both locally and internationally to provide committed support and resources to accelerate women’s empowerment and to ensure gender equality.

## Introduction

Intimate partner violence (IPV) is defined as any behaviour within an intimate relationship that causes physical, psychological, or sexual harm to those in the relationship.^1^^,^^2^ Considered as a violation of the Declaration of Human Rights,^3^ IPV is also a significant global public health issue. Although both men and women may be affected by IPV, it is worth recognizing that most incidents are perpetrated by men against women.^3^ Despite the emphasis of Sustainable Development Goal (SDG) 5.2 on the elimination of all forms of violence against women and girls,^4^ IPV remains alarmingly high. The 2021 WHO report indicated that approximately 30.0% of women aged 15–49 years worldwide have been subjected to physical or sexual violence by an intimate partner at least once in their lifetime.^5^ In addition, the WHO report showed that women in Africa and South-East Asia (33.0%) and those in low-income and middle-income countries (LMICs) were particularly vulnerable to violence due to environmental, economic, and cultural factors.^6^

IPV is a major problem with a wide range of short- and long-term implications for victims, including health issues such as bodily injuries, low self-esteem, depression, sexual dysfunction, anxiety, and post-traumatic stress disorder.^4^^,^^6^, ^7^, ^8^ Besides, existing research on IPV highlighted that it has deleterious effects on women’s education, employment and future relationships.^7^^,^^9^^,^^10^

IPV occurs across all social groups and stages of life. Even pregnant women are not spared. A comprehensive systematic review and meta-analysis estimated the global prevalence of intimate partner violence during pregnancy (IPVDP) to be approximately 9.2%.^11^ Based on evidence from 19 countries, the prevalence of IPVDP ranged from 2.0% to 13.5%.^12^ Further, a recent study conducted by Ahinkorah and colleagues^8^ found that about 6.0% of women in sub-Saharan Africa experienced IPVDP, compared with 13.6% in South Asia.^13^ Importantly, women who experience abuse during pregnancy are at an elevated risk for pregnancy-related complications, premature labour, ruptured spleen, low birth weight, and miscarriage.^12^^,^^14^ Similarly, foetuses/children exposed to IPVDP tend to be stunted, have low birth weights, and develop aggressive behaviours.^11^^,^^13^

Although several empirical studies suggest that low socioeconomic conditions amplify the risk of violence during pregnancy,^8^^,^^12^ IPVDP was under-documented in LMICs. The literature is mainly constituted by studies conducted in developed countries. Few existing studies on IPVDP in LMICs are restricted to specific countries or sub-regions, limiting generalizability. Besides, they focused more on factors associated with IPVDP, neglecting regional, sub-regional and cross-country disparities. Additionally, while efforts to combat IPV have increased in LMICs in recent decades, no study has investigated the evolving trends in IPVDP. Such investigation in IPVDP trends is necessary to carry out targeted interventions in countries with exacerbated prevalence and learn from best practices where substantial improvements have been achieved. Lastly, comparable population-based data on the prevalence of IPVDP in LMICs are lacking. To partially fill this gap, this comprehensive study aimed to examine national prevalence and disparities of IPVDP in 57 LMICs. Our study also explored changes in IPVDP and associated factors. Understanding the prevalence and risk factors related to IPVDP is crucial to develop appropriate prevention and mitigation policies and programs.

## Methods

### Study design and data sources

Data for this study were obtained from the Demographic and Health Survey (DHS) conducted in 57 low-income and middle-income countries (LMICs) between 2000 and 2024, grouped into six World Health Organization (WHO) regions.^15^ DHSs are nationally representative and cross-sectional household health surveys conducted regularly every 5 years in over 90 LMICs. Implemented with the collaboration of designated organizations such as Statistics offices, universities, ministries of health in participating countries, and the ICF Macro, DHSs were designed to provide information on health and demographic trends in developing countries. A two-stage stratified sampling design is applied in DHSs, involving the random selection of sample clusters created in the first stage, followed by the random selection of households within each cluster systematically using equal probabilities during the second stage.^16^ Further details on the DHS sampling are available elsewhere.^17^ Four questionnaires were designed to gather data: Household (HR), Women (IR), Men (MR), and Biomarker Questionnaire. A module related to domestic violence was included in the women’s questionnaire. The domestic violence module is routinely administered in a sub-sample of households where men were not interviewed. Only one woman currently or formerly in union per household was eligible for an interview about IPV. To ensure data quality and privacy, data were collected by trained interviewers in the absence of other household members.^17^ Note that a subset of countries collected data on IPV, and we selected those for which data were available.

### Ethics

The datasets employed were procured from the DHS program’s official repository website (https://dhsprogram.com). Informed consent was waived as the study utilized publicly available data. Given that this is a secondary analysis of publicly available data, no ethical approval was required. Moreover, the ethical approval had been received from the respective governments by the survey administration agencies.

### Population study and sample size

**Fig. 1:**
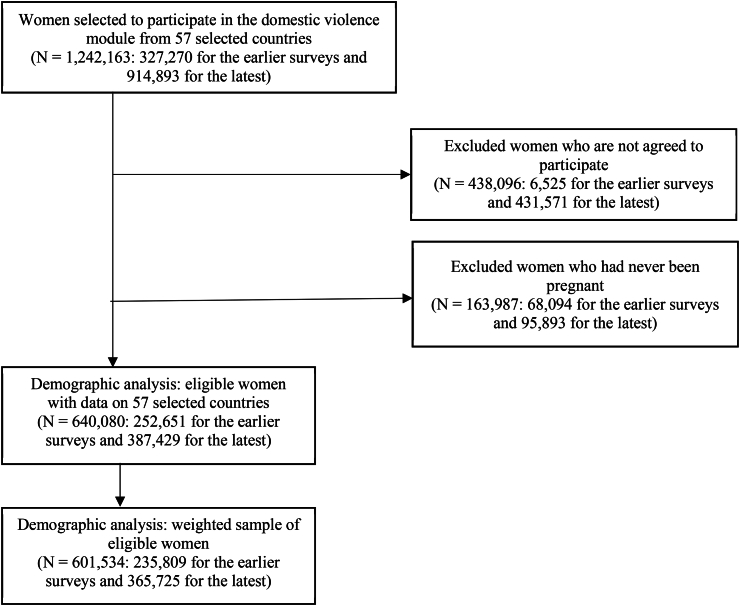
Flow diagram showing exclusions and final sample sizes of the study population. READ: For countries with at least two rounds of DHSs, the earlier surveys correspond to the oldest ones, and the latest to the most recent. A total of 1,242,163 women from 57 LMICs were selected to participate in the domestic violence module during 2000 and 2024. Importantly, 31 countries conducted at least two DHSs in this period, and 327,270 women were selected for the first round. In addition, 914,893 women were selected for the most recent DHS performed by the 57 LMICs.

To estimate the current prevalence and disparities of IPVDP, the latest DHS for each country was utilized, while to investigate changes in IPVDP countries with at least two rounds of DHSs between 2000 and 2024 were selected. Ultimately, our analysis included a weighted sample of 601,534 women aged 15–49 years from 57 countries (Fig. 1). The sample size for each respective country survey is reported in the Appendix Table S1 and Table S2.

### Outcome and selected explanatory variables

The outcome variable was IPVDP. Women who had been pregnant at any point in their lives were asked the following question: “*Has your husband/partner ever hit, slapped, kicked, or done anything else to hurt you physically while you were pregnant?*”. IPVDP was coded as dummy responses, with 1 for “Yes” and 0 for “No”. Other explanatory variables adjusted in our analysis included: women’s age, place of residence, level of education, wealth index, number of children, participation in household decision-making, age at first union, occupational status, and partner’s controlling behaviour (Appendix Table S3). Partner’s controlling behaviour and participation in household decision-making were composite variables (full coding can be found in Appendix Text S1). All selected socio-demographic variables were derived from a comprehensive literature review^7^^,^^8^^,^^15^^,^^18^^,^^19^ as well as their availability in the datasets.

### Statistics

We first merged the most recent DHS datasets of the 57 LMICs countries with the domestic violence module to estimate the current prevalence of IPVDP along with 95% CIs. Overall, regional, and sub-groups weighted prevalence of IPVDP were calculated by rescaling the sampling weights based on each country’s population size in the survey year,^20^ while the national prevalence of IPVDP was produced using the standard DHS weights of each country.

Second, to investigate disparities in IPVDP among different regions and across country income groupings, we used the six WHO Regions and the World Bank 2022 Income Classification (Low income, Lower-middle income, Upper-middle income).^21^ Detailed information about the classification is shown in Appendix Table S4.

Third, referring to the UNICEF technical note,^22^ we calculated the average annual rates of change (AARC) from earliest to latest surveys fitting a Poisson regression with robust error variance to explore changes in IPVDP. The equation is expressed as follows: y=α+βx+ε, where y=ln(PIPV), x=calendaryear, and β is the coefficient for calendar year. The AARC was reported as $100*\left[e^{\beta }-1\right]$, with a negative value to indicate a decrease and a positive value to indicate an increase in IPVDP. Countries with one DHS were excluded from the trend analysis.

Finally, using the latest DHSs, we examined the bivariate and multivariable factors associated with IPVDP. Pearson’s chi-square test, as part of the bivariate analysis, was used to assess significant relationships between the outcome (IPVDP) and each selected socio-demographic variable. A Poisson regression model was estimated to explore how selected socio-demographic variables were simultaneously associated with IPVDP (the model equation can be found in Appendix Text S2). Results were reported as adjusted rate ratios (aRR) with their 95% confidence intervals (CIs). Statistical significance was set at *p* < 0.05. We applied the variance inflation factor (VIF) to detect potential multicollinearity. All analyses were conducted using STATA (version 18) and R packages. The Strengthening the Reporting of Observational Studies in Epidemiology (STROBE) reporting guideline was followed.

### Role of the funding source

This study was not externally funded. The authors were individually supported by grants; however, these sponsors had no role in the study design, data collection, data analysis, data interpretation, writing or submitting this manuscript. Data supporting the findings of this study (pooled dataset) are available on reasonable request to the corresponding author.

## Results

This study included a total weighted sample of 601,534 women of childbearing age, with a mean age of 32.9 years (SD ± 8.6). Slightly more than 40.0% (40.6%) were from the African region, 22.3% from the South-East Asia, 20.4% from the region of the Americas, 6.9% from the Eastern Mediterranean region, 4.9% from the European region, and 4.8% from the Western Pacific (Appendix Table S5).

**Fig. 2:**
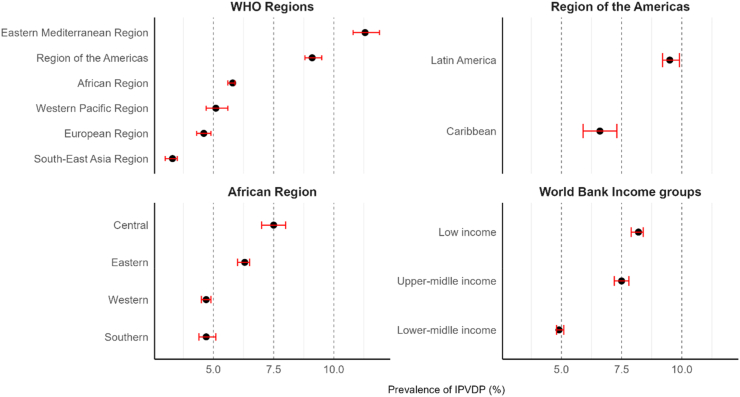
Prevalence of intimate partner violence during pregnancy (IPVDP) against women aged 15–49 years by WHO Regions, Sub-regions, and World Bank Income groups. IPVDP are presented as percentages (95% CIs).

Using the latest DHS of 57 LMICs, globally 6.3% (95% CI 6.2–6.4) women aged 15–49 years reported experiencing IPVDP. Fig. 2 and Appendix Table S6 showed variations across different WHO Regions. The highest prevalence of IPVDP was observed in the Eastern Mediterranean region (11.3% [95% CI 10.8–11.9]) and the lowest in the South-East Asia region (3.3% [95% CI 3.0–3.5]).

Further, there were sub-regional disparities (Fig. 2). For instance, in the region of the Americas, IPVDP was markedly higher in Latin America (9.5% [95% CI 9.2–9.9]) than in the Caribbean (6.6% [95% CI 5.9–7.3]). Besides, in the African region, the Central had the highest IPVDP (7.5% [95% CI 7.0–8.0]) and Southern (4.7% [95% CI 4.4–5.1]) and Western (4.7% [95% CI 4.5–4.9]) region, with similar prevalence, had the lowest. Additionally, there were important disparities in IPVDP by countries’ income levels (Fig. 2 and Appendix Table S7). Women from low-income countries faced the highest risk of IPVDP (8.2% [95% CI 7.9–8.4]), compared with 7.5% (95% CI 7.2–7.8) in upper-middle-income countries, and 4.9% (95% CI 4.8–5.1) in lower-middle-income countries.

**Fig. 3:**
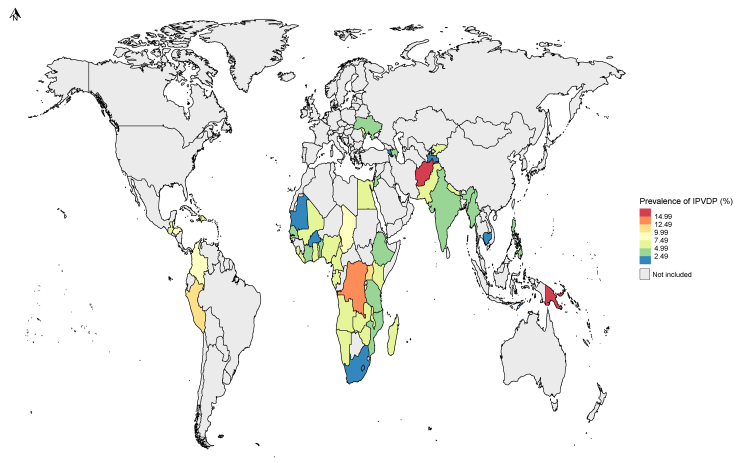
Prevalence of intimate partner violence during pregnancy (IPVDP) against women aged 15–49 years in 57 LMICs, using the latest DHSs. The full list of country prevalence estimates with their 95% CIs is shown in Appendix Table S8. Countries highlighted in grey are those for which data are unavailable.

IPVDP varied widely across countries, ranging from 1.1% to 17.6% in LMICs (Fig. 3 and Appendix Table S8). IPVDP was significantly highest in Papua New Guinea (17.6% [95% CI 15.3–20.0]; 2017), Afghanistan (15.7% [95% CI 14.8–16.6]; 2015), Democratic Republic of the Congo (12.5% [95% CI 11.2–13.8]; 2013), Peru (10.6% [95% CI 9.9–11.3]; 2012), and Uganda (10.6% [95% CI 9.7–11.4]; 2016); all exceeding 10.0%. By contrast, countries such as Burkina Faso (1.3% [95% CI 1.0–1.6]; 2021), Armenia (1.2% [95% CI 0.7–1.6]; 2016), Mauritania (1.2% [95% CI 0.7–1.6]; 2020), Tajikistan (1.2% [95% CI 0.8–1.6]; 2017) and South Africa (1.1% [95% CI 0.6–1.5]; 2016) had the lowest IPVDP, below 1.5%. Thus, Papua New Guinea recorded the highest IPVDP, and South Africa the lowest, with a difference of 16.5%.

In the African region, Democratic Republic of the Congo (12.5% [95% CI 11.2–13.8]; 2013) had the highest IPVDP and South Africa (1.1% [95% CI 0.6–1.5]; 2016) had the lowest prevalence (Fig. 3). In the Eastern Mediterranean, Afghanistan had the highest IPVDP at 15.7% (95% CI 14.8–16.6; 2015), and Jordan had the lowest occurrence of IPVDP at 2.7% (95% CI 1.9–3.5; 2023). In the European region, Kyrgyzstan had the highest IPVDP at 7.4% (95% CI 6.4–8.4; 2012), while both Tajikistan (1.2% [95% CI 0.8–1.6]; 2017) and Armenia (1.2% [95% CI 0.7–1.6]; 2016) reported the lowest. As for the region of the Americas, Peru had the highest IPVDP at 10.6% (95% CI 9.9–11.3; 2012), and Haiti had the lowest at 5.7% (95% CI 4.7–6.6; 2017). In South-East Asia, Nepal had the highest IPVDP at 5.9% (95% CI 5.0–6.8; 2022), whereas Timor-Leste had the lowest IPVDP occurrence at 2.3% (95% CI 1.6–2.9; 2016). Finally, in the Western Pacific region, Papua New Guinea (17.6% [95% CI 15.3–20.0]; 2017) had the highest IPVDP, and Cambodia had the lowest occurrence of IPVDP at 1.4% (95% CI 1.0–1.8; 2021).

Among the 10 countries with the highest IPVDP, 5 were in Africa and 3 in the region of the Americas (Appendix Table S8). Furthermore, of the 33 African countries in the study, more than one-third (n = 13) had a IPVDP of 6.5% or higher. Likewise, of the 4 Eastern Mediterranean countries, 3 (Afghanistan [2015], Pakistan [2018], and Egypt [2014]) recorded a IPVDP above 6.5%. In the European region, except for Kyrgyzstan [2012] and Moldova [2005], the other 4 countries (Azerbaijan [2006], Ukraine [2007], Armenia [2016], and Tajikistan [2017]) had IPVDPs below 5.0%. Among the 6 countries in the region of the Americas, except for Haiti [2017], 5 had a IPVDP of 7.0% and higher (Peru [2012], Colombia [2015], Honduras [2012], Dominican Republic [2013], and Guatemala [2015]), all located in Latin America. As for the 5 South-East Asian countries, only Nepal [2022] had IPVDP higher than 5.0%, while the IPVDP of the 4 other countries (Maldives [2017], Myanmar [2016], India [2019], and Timor-Leste [2016]) ranged from 2.0–4.0%. Concerning the 3 Western Pacific countries, except for Papua New Guinea [2017], Philippines [2022] and Cambodia [2021] had IPVDPs ranging from 1.0–3.0%.

**Fig. 4:**
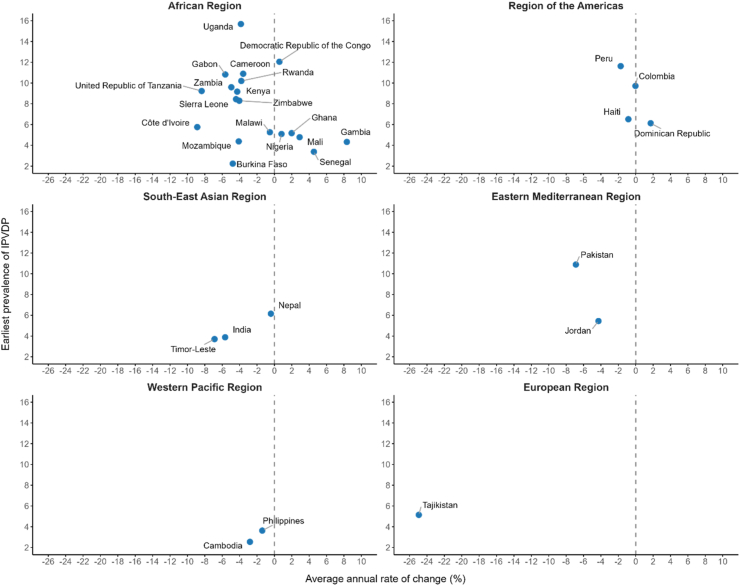
Earliest prevalence of IPVDP and AARC in 31 countries categorised by WHO Regions. AARC = average annual rate of change. The AARCs and their 95% CIs for all countries were presented in the Appendix Table S9. Notes: A negative AARC value indicates a reduction in the prevalence of IPVDP, and a positive value, an increase.

The trends in IPVDP in each country were shown in Fig. 4, Appendix Table S9 and Appendix Fig. S1. The AARC in 31 countries indicated that three-quarters (23 [74.2%] of 31) experienced a decreasing trend in IPVDP. The largest decline was observed in Tajikistan (−24.9% [95% CI –25.6 to −24.2]; 2012–2017), Côte d’Ivoire (−8.9% [95% CI –9.8 to −8.0]; 2012–2021), and Tanzania (−8.4% [95% CI –9.4 to −7.3]; 2010–2022). Conversely, Nepal (−0.4% [95% CI –1.5 to 0.7]; 2011–2022), Malawi (−0.5% [95% CI –1.4 to 0.3]; 2004–2015) and Haiti (−0.8% [95% CI –1.9 to 0.2]; 2000–2017) had the smallest declining trends. There were 7 countries with significantly positive AARC of change, with the largest of 8.3% (95% CI 6.8–9.9; 2013–2020) in Gambia, followed by 4.5% (95% CI 3.1–6.0; 2017–2019) in Senegal, and 2.9% (95% CI 1.8–4.0; 2006–2018) in Mali. Colombia was the only country that has not shown any change (2005–2015), since the IPVDP prevalence of earliest and latest surveys was similar. Furthermore, around a third (6 [31.6%] of 19) of African countries exhibited increasing trends in IPVDP. Only the Dominican Republic presented increased IPVDP prevalence in the region of the Americas, with an AARC of 1.7% (95% CI 0.8–2.7; 2002–2013). Countries from South-East Asian, European, Eastern Mediterranean, and Western Pacific regions all showed reduced prevalence of IPVDP; the country with the most significant reduction (Tajikistan) was in Europe.

**Fig. 5:**
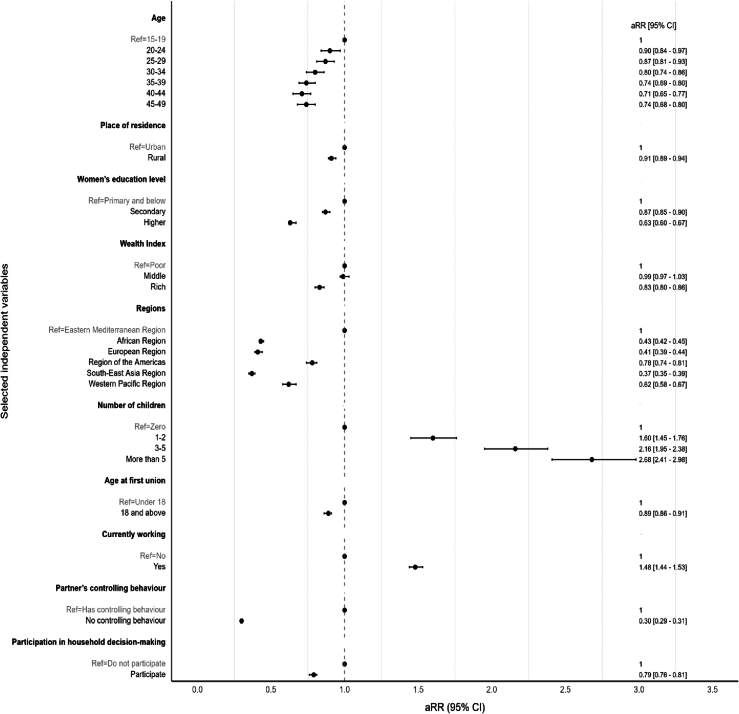
Association between IPVDP and selected independent variables in 57 LMICs using the latest surveys. Reference categories (Ref) are shown in grey. Data are adjusted risk ratios (95% CI)–an adjusted risk ratio less than 1 indicates a decreased risk of experiencing IPVDP; an adjusted risk ratio greater than 1 indicates an increased risk of experiencing IPVDP. The prevalence is adjusted to women’s age, place of residence, education level, wealth index, regions, number of children, age at first union, occupational status, partner’s controlling behaviour, and participation in household decision-making.

Bivariate associations of correlates indicated that IPVDP was most prevalent among women: aged 15–19 years (7.1%); from rural areas (6.4%); with primary education and below (7.5%); from poor households (7.2%); who had not participated in household decision-making (8.1%); who had more than 5 children (9.2%); who had an income-generating activity (6.9%); who had their first union under 18 years of age (7.5%); and whose partners exhibited controlling behaviour (8.9%) (Appendix Table S10). The results of the multivariable regression model also revealed that women aged 20–24 (aRR = 0.90; 95% CI 0.84–0.97; *p* = 0.005), 25–29 (aRR = 0.87; 95% CI 0.81–0.93; *p* < 0.001), 30–34 (aRR = 0.80; 95% CI 0.74–0.86; *p* < 0.001), 35–39 (aRR = 0.74; 95% CI 0.69–0.80; *p* < 0.001), 40–44 (aRR = 0.71; 95% CI 0.65–0.77; *p* < 0.001), and 45–49 (aRR = 0.74; 95% CI 0.68–0.80; *p* < 0.001) had lower likelihood of IPVDP compared to those aged 15–19 (Fig. 5). Similarly, participants from the African region (aRR = 0.43; 95% CI 0.42–0.45; *p* < 0.001), European region (aRR = 0.41; 95% CI 0.39–0.44; *p* < 0.001), region of the Americas (aRR = 0.78; 95% CI 0.74–0.81; *p* < 0.001), South-East region (aRR = 0.37; 95% CI 0.35–0.39; *p* < 0.001), and Western Pacific region (aRR = 0.62; 95% CI 0.58–0.67; *p* < 0.001) had lower likelihood of IPVDP compared to those from the Eastern Mediterranean region. Being from rural areas (aRR = 0.91; 95% CI 0.89–0.94; *p* < 0.001) decreased the risk of IPVDP. The likelihood of IPVDP was 13.0% and 37.0% respectively lower among women with secondary (aRR = 0.87; 95% CI 0.85–0.90; *p* < 0.001) and higher educational level (aRR = 0.63; 95% CI 0.60–0.67; *p* < 0.001) than those with primary level and below. Additionally, women from rich households had a lower likelihood of IPVDP (aRR = 0.83; 95% CI 0.80–0.86; *p* < 0.001) compared to those from poor households. The results displayed that women who had 1-2 children (aRR = 1.60; 95% CI 1.45–1.76; *p* < 0.001), 3–5 children (aRR = 2.16; 95% CI 1.95–2.38; *p* < 0.001), more than 5 children (aRR = 2.68; 95% CI 2.41–2.98; *p* < 0.001), and women who had an income-generating activity (aRR = 1.48; 95% CI 1.44–1.53; *p* < 0.001) had the strongest association with IPVDP compared to their counterparts. Getting married at 18 and above (aRR = 0.89; 95% CI 0.86–0.91; *p* < 0.001) was associated with decreased risks of experiencing IPVDP. Moreover, IPVDP was less likely to occur among women who had participated in household decision-making (aRR = 0.79; 95% CI 0.76–0.81; *p* < 0.001) and those whose partners exhibited controlling behaviour (aRR = 0.30; 95% CI 0.29–0.31; *p* < 0.001).

## Discussion

To our knowledge, the current study was the first to examine prevalence, disparities, trends in IPVDP, and factors associated with this phenomenon in 57 LMICs. The overall prevalence of IPVDP was estimated at 6.3% (95% CI 6.2–6.4), which suggests that it is a common undesirable experience. Importantly, this prevalence masked significant regional, sub-regional, and national disparities. IPVDP also varied by countries’ income levels and increased considerably in some countries. Lastly, we found that IPVDP was associated with various social-ecological factors.

Our findings highlighted that the Eastern Mediterranean recorded the highest prevalence of IPVDP (11.3% [95% CI 10.8–11.9]) among the six WHO Regions. Socio-cultural factors influenced this elevated prevalence in the Eastern Mediterranean region. Countries in this region share many socio-cultural characteristics in common, including the “silence” about IPV.^15^^,^^23^ According to Abadeer,^24^ inherited social norms and traditions in most countries in this region condone and even encourage IPV. For instance, in Egypt, Pakistan, and other settings, laws rooted in societal norms provide justification for men’s violent behaviours against women, framing them as a response to perceived dishonour resulting from a woman’s violation of established social and moral values.^25^ This is known as “honour crimes”.^24^ In addition, national family laws in many countries in the Eastern Mediterranean provide an inferior legal status for women, giving men more privileges and complete authority to abuse, manipulate and control them.^25^

Our study demonstrated sub-regional disparities, with the magnitude of IPVDP being much higher in Latin America (in the region of the Americas) and Central Africa (in the African region). Fineran and colleagues^26^ argue that the Latinx culture is “embedded in gender inequality”, which is the root cause of IPV. Women from Latin American are oppressed by powerful and rigid patriarchal social structures (machismo).^27^ Men display symbolic and practical dominance over their partners. Further, Latin American communities adhere to collectivist norms i.e., women are expected to accept IPV as fate and endure the experience in silence to preserve the family from shame.^26^ Respect for the cultural dominance of machismo is often used by perpetrators as a resource to silence victims and prevent them from seeking assistance. As for Central Africa, the observed high IPVDP compared to Eastern, Western and Southern African regions can be partly attributed to the various conflicts that countries in this sub-region have experienced in recent decades. As Bergenfeld and colleagues^28^ emphasize, IPV tends to increase following conflict situations due to their challenges (financial stress, decline in mental health), risk factors for increased tension within couples.

The findings indicate that IPVDP was most prevalent among women from low-income countries (8.2% [95% CI 7.9–8.4]), where structural inequalities and socioeconomic barriers exacerbate their vulnerability. Although a country’s income level is not a direct causal factor for IPVDP, it is an indicator of broader social processes that accompany economic development, including education, employment opportunities, and gender norms.^10^^,^^29^ Women in low-income countries commonly have limited access to education due to inadequate public investment in girls’ schooling, which in turn constrains their ability to secure skilled or paid employment. The resulting economic dependence further reinforces social hierarchies, which limit their autonomy and increase the risk of experiencing IPVDP.^4^^,^^7^ Additionally, living in marginalized contexts characterized by resource limitations perpetuates gender inequality and entrenches patriarchal norms, particularly the belief in male superiority, which further legitimizes and perpetuates cycles of intimate partner violence.^10^^,^^30^

Our findings also revealed IPVDP was significantly decreasing in many studied countries. After the 1994 International Conference on Population and Development (ICPD) in Cairo under the auspices of the United Nations, efforts to prevent IPV in all its forms have intensified in LMICs, including the adoption of legislation, raising awareness about IPV as well as its consequences, and the creation and improvement of social services to care for IPV victims and their children.^31^ Ning and colleagues^15^ reported that at least 118 LMICs have implemented laws against IPV in recent decades and have been using WHO guidelines to adopt interventions and national guidelines on violence against women and girls. An example is Kenya, where policy frameworks and programmatic guidance were developed towards enabling legislation on gender-based violence services and with public health facilities, causing a decrease in IPV.^31^ Nigeria achieved a reduction in IPV through its investment in financial and reproductive literacy, which promoted gender equality, and relationship equality among couples.^32^ Note further that despite the growing number of initiatives aimed at eliminating discrimination against women, positive AARC has been observed in some countries such as Democratic Republic of the Congo, Gambia and Mali; all of which have been heavily affected by repeated armed conflicts in the past years.^33^ As previously mentioned, challenges of the conflicts, including poverty and lack of income generating opportunities which affect men’s social standing– all significantly contributed to the heightened risk of IPVDP.^15^

Although the prevalence of IPVDP declined in most LMICs, it was still high in countries like Uganda, Burundi, Chad, Togo, Cameroon, Gabon, Ghana, Kenya, Peru, Sierra Leone, and Papua New Guinea. In addition to socio-political and economic instability, women from these countries have a strong tendency to justify men’s wife beating.^6^^,^^19^ For example, a recent study revealed that about 70.0% of women in Papua New Guinea justified physical IPV.^34^ In such environments, IPV is accepted as normal behaviour where women are less likely to confront their perpetrators, which in turn aggravates the cycle of IPV.^3^ Similarly, Many of these countries are characterized by high rates of child marriage.^7^ Child marriage exposes girls to harsh realities that they are not mature enough to handle, forces them to drop out of school, thereby increasing the risks of IPV as they are in power imbalanced marriages.^3^ Besides, women from these countries are marginalized in all spheres of society due to traditional gender and societal norms,^34^ keeping them in widespread poverty. Conversely, South Africa was the country with the lowest prevalence of IPVDP. A program called “Stepping Stones” has been set up in South Africa, which addresses conflict within intimate relationships and cultivates mutual understanding of partners, reducing the prevalence of physical IPV and forced sex.^34^^,^^35^ Other countries could learn from South Africa’s initiative to significantly reduce their prevalence of IPVDP.

Across examples of successful reductions in IPVDP, are policies and strategies, including robust legislative frameworks,^36^ health system integration of IPV identification and support,^37^ and community-driven normative change through economic empowerment and participatory programming.^38^ These efforts ensure the utilization of multi-level, cross-sectoral levers of change that are essential to driving down IPVDP, and offer a clear roadmap for policymakers and practitioners aiming to replicate these gains in other similar contexts.

In analysing factors associated with IPVDP, we found that disadvantaged groups (i.e., 15–19 years old, poor, women with primary education and below, women who had more than 5 children, those married at under 18, women who had not participated in household decision-making, and those whose partners exhibited controlling behaviour) had higher likelihood of experiencing IPVDP. This observation is in agreement with other studies.^8^^,^^11^ In LMICs, these groups of women are extremely vulnerable, have very limited perspectives for the future, and exhibited a higher tendency to justify IPV.^6^^,^^7^^,^^10^ These factors compromise women’s economic independence and empowerment, and represent critical pathways for IPVDP.^7^ They further constitute potential barriers to victims leaving and terminating relationships with perpetrators.^4^ Supported by a multi-country study,^8^ our findings also suggest that women from rural areas had lower likelihood of IPVDP compared to their counterparts from urban areas. Growing urbanization in LMICs is accompanied by major social and economic difficulties that can generate stressful living conditions, which raise the risk of women’s IPV.^4^ Besides, the multivariate results indicated that women with an income-generating activity had a higher likelihood of IPVDP compared to those without any income-generating activity, which is congruent with previous findings.^3^^,^^15^ Vyas and Watts^39^ suggest that economically disadvantaged men may resort to abusive behaviours to assert control over their employed female partners, viewing their financial independence as a challenge to their masculinity and authority. Similarly, other researchers argue that women who engage in income-generating activities may face an increased risk of violence, as their financial autonomy could be perceived as a direct threat to their partner’s dominance within the household.^3^

This study had several strengths. It was the first study to have examined IPVDP in 57 LMICs with substantial sample sizes covering all WHO geographical regions. The study also offered a general understanding of how IPVDP has changed over the past years in many countries, and this might help to develop targeted interventions. Another key strength was that DHSs use standard procedures and validated questionnaires designed to enable comparison at national and regional levels. Additionally, rigorous analytical methods were used to minimize sampling errors, which has enabled us to obtain reliable conclusions. However, this study was not free of limitations. First, it involved 57 LMICS for which IPVDP data was available, implying that it was not representative of all LMICs. Furthermore, the countries within each global geographical region do not form representative samples of all LMICs in those regions. As a result, despite their robustness and broad geographical coverage, the findings of this study are not generalisable to LMICs at the regional or global level. However, an exception can be made for sub-Saharan Africa and South Asia, where the countries included are undoubtedly representative of the regions. Consequently, the IPVDP statistics estimated by this study for countries in sub-Saharan Africa and South Asia are generalisable and representative of the level of IPVDP in these regions. Second, given that violence is a sensitive topic, and the study was limited to physical violence during pregnancy, the prevalence of IPVDP may be under-reported/estimated, thereby skewing the results. Third, the study was limited using secondary data, restricting study variables. Fourth, since two survey rounds were used to calculate the AARC, the trend analysis in IPVDP contained bias, ignoring changes occurring within the period. Fifth, note that 26 countries were excluded from the trend analysis. Sixth, irregular time intervals in the surveys across countries biased the comparison trends across countries. Lastly, owing to the nature of the cross-sectional study design, we are unable to infer causality in the relationships among various independent variables and IPVDP.

This study comprehensively analyses the prevalence and factors associated with IPVDP across 57 LMICs. Findings reveal significant regional and socioeconomic disparities. While progress has been achieved through policy interventions, legal frameworks, and gender-equality initiatives, persistent socio-cultural norms, economic challenges, and conflict-related stressors continue to drive IPVDP in many regions. Our findings emphasize the need for targeted, context-specific interventions that address structural inequalities, promote financial independence for women, and challenge harmful societal norms toward IPV.^40^ Enforcing and strengthening legal protections, improving access to education, and fostering community-driven prevention strategies are essential to reducing IPVDP and advancing gender equality globally. Further, increasing awareness and support services for survivors, particularly in regions with high prevalence rates, can help mitigate the long-term consequences of IPV during pregnancy and improve maternal and child health outcomes.^41^

## Contributors

Conception and design: DJS, and VCKT. Literature review: DJS, AK, and CZO. Data management and analysis: DJS, VCKT, AO, and KD. Interpretation of the results: DJS, CZO, and VCKT. Drafting of the article: DJS, VCKT, AO, KD, AK, CZO, and BP. Review, editing, and supervision: DJS, VCKT, AO, KD, AK, CZO, and BP. All authors had full access to all the data in the study and had final responsibility for the decision to submit for publication. All authors read and approved the final version of the manuscript.

## Data sharing statement

The data used in this study is publicly available at: https://dhsprogram.com/data/availabledatasets.cfm.

## Editor note

The Lancet Group takes a neutral position with respect to territorial claims in published maps and institutional affiliations.

## Declaration of interests

The authors declare that they have no competing interests.
