# Supplementary material for: Intimate partner violence during pregnancy against 601,534 women aged 15 to 49 years in 57 LMICs: prevalence, disparities, trends and associated factors using Demographic and Health Survey data
Source: eClinicalMedicine. 2025 Jul 24;86:103382. doi: 10.1016/j.eclinm.2025.103382 (PMC12312060; doi:10.1016/j.eclinm.2025.103382)
Supplement: Appendix Fig. S1 and Tables S1–S10 [file mmc1.docx]

**Supplemental materials**

Table S1: Weighted sample size of eligible women by country, using the earliest survey (Data were only available for 31 countries)

| **Survey year** | **Countries** | **N** | **Percentage** |
| --- | --- | --- | --- |
| 2010 | Burkina Faso | 9244 | 3,9 |
| 2000 | Cambodia | 2288 | 1,0 |
| 2004 | Cameroon | 2441 | 1,0 |
| 2005 | Colombia | 27119 | 11,5 |
| 2012 | Côte d'Ivoire | 5093 | 2,2 |
| 2007 | Democratic Republic of the Congo | 2602 | 1,1 |
| 2002 | Dominican Republic | 6630 | 2,8 |
| 2012 | Gabon | 4250 | 1,8 |
| 2013 | Gambia | 3128 | 1,3 |
| 2008 | Ghana | 1761 | 0,7 |
| 2000 | Haiti | 2264 | 1,0 |
| 2016 | India | 57842 | 24,5 |
| 2007 | Jordan | 3213 | 1,4 |
| 2014 | Kenya | 4331 | 1,8 |
| 2004 | Malawi | 8008 | 3,4 |
| 2006 | Mali | 7964 | 3,4 |
| 2011 | Mozambique | 5643 | 2,4 |
| 2011 | Nepal | 2982 | 1,3 |
| 2008 | Nigeria | 16396 | 7,0 |
| 2012 | Pakistan | 3395 | 1,4 |
| 2006 | Peru | 21498 | 9,1 |
| 2008 | Philippines | 6747 | 2,9 |
| 2005 | Rwanda | 2593 | 1,1 |
| 2017 | Senegal | 2277 | 1,0 |
| 2013 | Sierra Leone | 4390 | 1,9 |
| 2012 | Tajikistan | 3834 | 1,6 |
| 2009 | Timor-Leste | 1905 | 0,8 |
| 2006 | Uganda | 1643 | 0,7 |
| 2010 | United Republic of Tanzania | 5515 | 2,3 |
| 2007 | Zambia | 4140 | 1,8 |
| 2005 | Zimbabwe | 4674 | 2,0 |
|  | **Total** | **235809** | **100,0** |

Table S2: Weighted sample size of eligible women by country, using the latest survey (Data were available for 57 countries)

| **Survey year** | **Countries** | **N** | **Percentage** |
| --- | --- | --- | --- |
| 2015 | Afghanistan | 20119 | 5,5 |
| 2015 | Angola | 10696 | 2,9 |
| 2016 | Armenia | 3069 | 0,8 |
| 2006 | Azerbaijan | 3615 | 1,0 |
| 2018 | Benin | 4237 | 1,2 |
| 2021 | Burkina Faso | 8301 | 2,3 |
| 2016 | Burundi | 6775 | 1,9 |
| 2021 | Cambodia | 5224 | 1,4 |
| 2018 | Cameroon | 4896 | 1,3 |
| 2015 | Chad | 3533 | 1,0 |
| 2015 | Colombia | 25731 | 7,0 |
| 2012 | Comoros | 1882 | 0,5 |
| 2021 | Côte d'Ivoire | 3852 | 1,1 |
| 2013 | Democratic Republic of the Congo | 5269 | 1,4 |
| 2013 | Dominican Republic | 5169 | 1,4 |
| 2014 | Egypt | 6406 | 1,8 |
| 2016 | Ethiopia | 4207 | 1,2 |
| 2020 | Gabon | 3112 | 0,9 |
| 2020 | Gambia | 1692 | 0,5 |
| 2022 | Ghana | 4226 | 1,2 |
| 2015 | Guatemala | 5905 | 1,6 |
| 2017 | Haiti | 4036 | 1,1 |
| 2012 | Honduras | 11368 | 3,1 |
| 2019 | India | 59028 | 16,1 |
| 2023 | Jordan | 5237 | 1,4 |
| 2022 | Kenya | 12610 | 3,4 |
| 2012 | Kyrgyzstan | 4169 | 1,1 |
| 2024 | Lesotho | 1753 | 0,5 |
| 2019 | Liberia | 2533 | 0,7 |
| 2021 | Madagascar | 5745 | 1,6 |
| 2015 | Malawi | 5102 | 1,4 |
| 2017 | Maldives | 2723 | 0,7 |
| 2018 | Mali | 3157 | 0,9 |
| 2020 | Mauritania | 3093 | 0,8 |
| 2022 | Mozambique | 3956 | 1,1 |
| 2016 | Myanmar | 2797 | 0,8 |
| 2013 | Namibia | 1652 | 0,5 |
| 2022 | Nepal | 3804 | 1,0 |
| 2018 | Nigeria | 8349 | 2,3 |
| 2018 | Pakistan | 3044 | 0,8 |
| 2017 | Papua New Guinea | 3509 | 1,0 |
| 2012 | Peru | 13152 | 3,6 |
| 2022 | Philippines | 11319 | 3,1 |
| 2005 | Republic of Moldova | 3943 | 1,1 |
| 2020 | Rwanda | 1849 | 0,5 |
| 2008 | São Tome and Principe | 1647 | 0,5 |
| 2019 | Senegal | 1275 | 0,3 |
| 2019 | Sierra Leone | 4045 | 1,1 |
| 2016 | South Africa | 3769 | 1,0 |
| 2017 | Tajikistan | 4703 | 1,3 |
| 2016 | Timor-Leste | 3196 | 0,9 |
| 2014 | Togo | 5064 | 1,4 |
| 2016 | Uganda | 7114 | 1,9 |
| 2007 | Ukraine | 2172 | 0,6 |
| 2022 | United Republic of Tanzania | 3875 | 1,1 |
| 2018 | Zambia | 7416 | 2,0 |
| 2015 | Zimbabwe | 5603 | 1,5 |
|  | **Total** | **365725** | **100,0** |

Table S3: Selected explanatory variables and their categories

| **Other explanatory variables** | **Categories** |  |  |  |
| --- | --- | --- | --- | --- |
|  |  |  |  |  |
| **Age** |  |  |  |  |
|  | 15-19 |  |  |  |
|  | 20-24 |  |  |  |
|  | 25-29 |  |  |  |
|  | 30-34 |  |  |  |
|  | 35-39 |  |  |  |
|  | 40-44 |  |  |  |
|  | 45-49 |  |  |  |
| **Place of residence** |  |  |  |  |
|  | Urban |  |  |  |
|  | Rural |  |  |  |
| **Women's education level** |  |  |  |  |
|  | Primary and below |  |  |  |
|  | Secondary |  |  |  |
|  | Higher |  |  |  |
| **Wealth Index** |  |  |  |  |
|  | Poor |  |  |  |
|  | Middle |  |  |  |
|  | Rich |  |  |  |
| **Regions** |  |  |  |  |
|  | African Region |  |  |  |
|  | Eastern Mediterranean Region | | |  |
|  | European Region |  |  |  |
|  | Region of the Americas |  |  |  |
|  | South-East Asia Region |  |  |  |
|  | Western Pacific Region |  |  |  |
| **Number of children** |  |  |  |  |
|  | Zero |  |  |  |
|  | 1-2 |  |  |  |
|  | 3-5 |  |  |  |
|  | More than 5 |  |  |  |
| **Age at first union** |  |  |  |  |
|  | Under 18 |  |  |  |
|  | 18 and above |  |  |  |
| **Currently working** |  |  |  |  |
|  | No |  |  |  |
|  | Yes |  |  |  |
| **Partner’s controlling behaviour** |  |  |  |  |
|  | No controlling behaviour |  |  |  |
|  | Has controlling behaviour |  |  |  |
| **Participation in household decision-making** |  |  |  |  |
|  | No |  |  |  |
|  | Yes |  |  |  |

Note: Other variables such as the partner's education, occupation and alcohol consumption could also be considered. We decided to exclude them due to many missing data, which would bias our results.

----------------

Text S1: Construction of the composite variables ' Partner’s controlling behaviour' and 'Women’s participation in household decision-making'

**Stata code for the construction of the ‘Partner’s controlling behaviour’ composite variable**

gen controle=d101a+d101b+d101c+d101d+d101e+d101f

replace controle=1 if controle>=1

lab def controle 0"No" 1"Yes"

lab val controle controle

variable name variable label

-----------------------------------------------------------------------------------------------------------------

d101a husband/partner jealous if respondent talks with other men

d101b husband/partner accuses respondent of unfaithfulness

d101c husband/partner does not permit respondent to meet female friends

d101d husband/partner tries to limit respondent's contact with family

d101e husband/partner insists on knowing where respondent is

d101f husband/partner doesn't trust respondent with money

**Stata code for the construction of the** **'Women’s participation in household decision-making' composite variable**

gen health=1 if v743a==1|v743a==2

replace health=0 if health>=2

gen hous=1 if v743b==1|v743b==2

replace hous=0 if hous>=2

gen visit=1 if v743d==1|v743d==2

replace visit=0 if visit>=2

gen decision=health+hous+visit

replace decision=1 if decision>=1

lab def decision 1"Yes" 0"No"

lab val decision decision

variable name variable label

-----------------------------------------------------------------------------------------------------------------

v743a person who usually decides on respondent's health care

v743b person who usually decides on large household purchases

v743d person who usually decides on visits to family or relatives

Table S4: Country classification by WHO Regions and income levels

| **No** | **Pays** | **WHO Regions** | **World Bank ranking of income** |
| --- | --- | --- | --- |
| 1 | Afghanistan | Eastern Mediterranean Region | Low |
| 2 | Angola | African Region | Lower middle |
| 3 | Armenia | European Region | Upper middle |
| 4 | Azerbaijan | European Region | Upper middle |
| 5 | Benin | African Region | Lower middle |
| 6 | Burkina Faso | African Region | Low |
| 7 | Burundi | African Region | Low |
| 8 | Cambodia | Western Pacific Region | Lower middle |
| 9 | Cameroon | African Region | Lower middle |
| 10 | Chad | African Region | Low |
| 11 | Colombia | Region of the Americas | Upper middle |
| 12 | Comoros | African Region | Lower middle |
| 13 | Côte d'Ivoire | African Region | Lower middle |
| 14 | Democratic Republic of the Congo | African Region | Low |
| 15 | Dominican Republic | Region of the Americas | Upper middle |
| 16 | Egypt | Eastern Mediterranean Region | Lower middle |
| 17 | Ethiopia | African Region | Low |
| 18 | Gabon | African Region | Upper middle |
| 19 | Gambia | African Region | Low |
| 20 | Ghana | African Region | Lower middle |
| 21 | Guatemala | Region of the Americas | Upper middle |
| 22 | Haiti | Region of the Americas | Low |
| 23 | Honduras | Region of the Americas | Lower middle |
| 24 | India | South-East Asia Region | Lower middle |
| 25 | Jordan | Eastern Mediterranean Region | Upper middle |
| 26 | Kenya | African Region | Lower middle |
| 27 | Kyrgyzstan | European Region | Lower middle |
| 28 | Lesotho | African Region | Lower middle |
| 29 | Liberia | African Region | Low |
| 30 | Madagascar | African Region | Low |
| 31 | Malawi | African Region | Low |
| 32 | Maldives | South-East Asia Region | Upper middle |
| 33 | Mali | African Region | Low |
| 34 | Mauritania | African Region | Lower middle |
| 35 | Mozambique | African Region | Low |
| 36 | Myanmar | South-East Asia Region | Lower middle |
| 37 | Namibia | African Region | Upper middle |
| 38 | Nepal | South-East Asia Region | Lower middle |
| 39 | Nigeria | African Region | Lower middle |
| 40 | Pakistan | Eastern Mediterranean Region | Lower middle |
| 41 | Papua New Guinea | Western Pacific Region | Lower middle |
| 42 | Peru | Region of the Americas | Upper middle |
| 43 | Philippines | Western Pacific Region | Lower middle |
| 44 | Moldova | European Region | Lower middle |
| 45 | Rwanda | African Region | Low |
| 46 | Senegal | African Region | Lower middle |
| 47 | Sierra Leone | African Region | Low |
| 48 | South Africa | African Region | Upper middle |
| 49 | Sao Tome and Principe | African Region | Lower middle |
| 50 | Tajikistan | European Region | Low |
| 51 | Timor-Leste | South-East Asia Region | Lower middle |
| 52 | Togo | African Region | Low |
| 53 | Uganda | African Region | Low |
| 54 | Ukraine | European Region | Lower middle |
| 55 | Tanzania | African Region | Lower middle |
| 56 | Zambia | African Region | Lower middle |
| 57 | Zimbabwe | African Region | Lower middle |

Note: We used the World Bank 2022 Income Classification

----------------------------

Text S2: The Poisson model equation

Poisson regression is a regression analysis for count and rate data. (<https://online.stat.psu.edu/stat462/node/209/>).


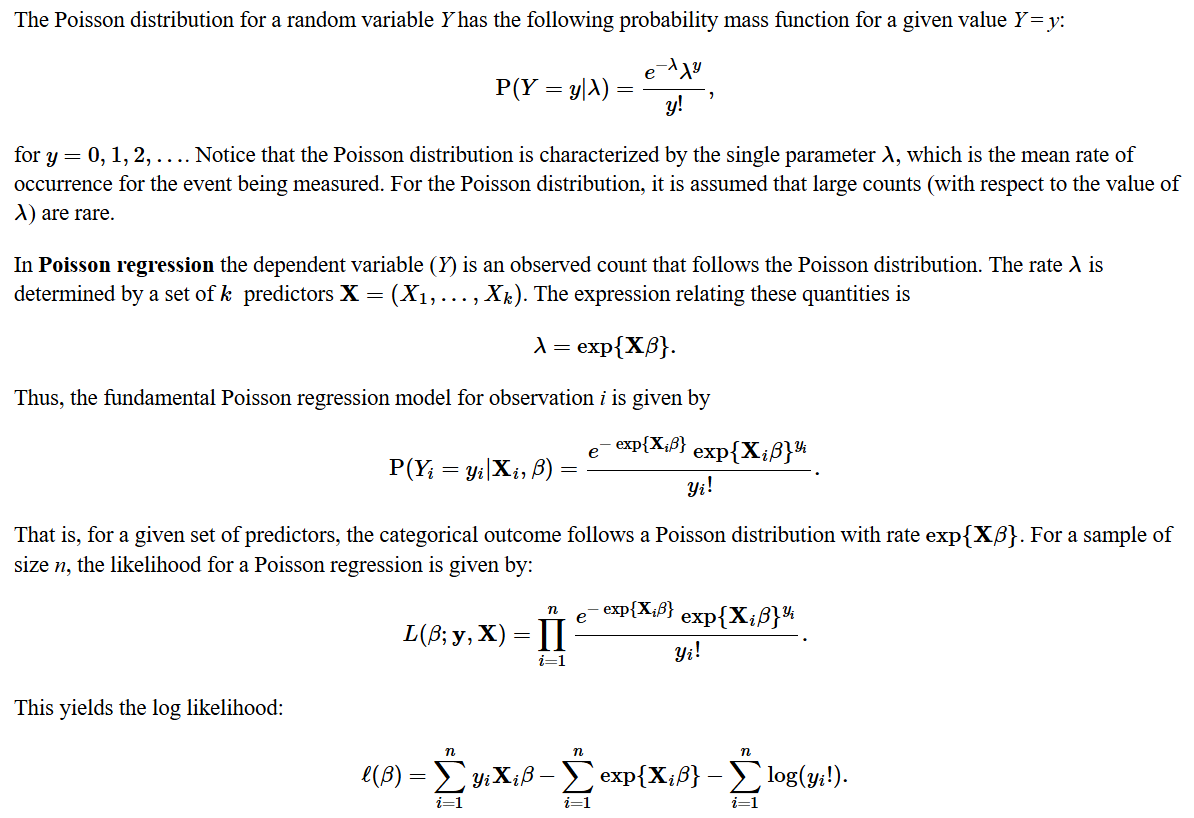


Table S5: Socio-demographic characteristics of the study population

| **Socio-demographic  characteristics** | **Women aged 15-49 years** | |
| --- | --- | --- |
|  | N (%) Unweighted | N (%) Weighted |
| **Age** |  |  |
| 15-19 | 28096 (4.4) | 27764 (4.6) |
| 20-24 | 94992 (14.8) | 90610 (15.1) |
| 25-29 | 128829 (20.1) | 114040 (19.0) |
| 30-34 | 124605 (19.5) | 108970 (18.1) |
| 35-39 | 108021 (16.9) | 100954 (16.8) |
| 40-44 | 83756 (13.1) | 84167 (14.0) |
| 45-49 | 71781 (11.2) | 75030 (12.5) |
| **Place of residence** |  |  |
| Urban | 252405 (39.4) | 259829 (43.2) |
| Rural | 387675 (60.6) | 341705 (56.8) |
| **Women's education level** |  |  |
| Primary and below | 354749 (55.4) | 321272 (53.4) |
| Secondary | 219093 (34.2) | 212770 (35.4) |
| Higher | 66238 (10.3) | 67491 (11.2) |
| **Regions** |  |  |
| African Region | 261808 (40.9) | 244333 (40.6) |
| Eastern Mediterranean Region | 42464 (6.6) | 41415 (6.9) |
| European Region | 32128 (5.0) | 29551 (4.9) |
| Region of the Americas | 129654 (20.3) | 122871 (20.4) |
| South-East Asia Region | 142360 (22.2) | 134277 (22.3) |
| Western Pacific Region | 31666 (4.9) | 29087 (4.8) |
| **Number of children** |  |  |
| Zero | 21425 (3.3) | 22205 (3.7) |
| 1-2 | 280649 (43.8) | 271020 (45.1) |
| 3-5 | 247370 (38.6) | 223123 (37.1) |
| More than 5 | 90636 (14.2) | 85186 (14.2) |
| **Age at first union** |  |  |
| Under 18 | 246009 (38.4) | 230095 (38.3) |
| 18 and above | 394071 (61.6) | 371438 (61.7) |
| **Currently working** |  |  |
| No | 263468 (41.2) | 244164 (40.6) |
| Yes | 376612 (58.8) | 357370 (59.4) |
| **Partner’s controlling behaviour** |  |  |
| No controlling behaviour | 250509 (39.1) | 229973 (38.2) |
| Has controlling behaviour | 389571 (60.9) | 371561 (61.8) |
| **Participation in household decision-making** |  |  |
| No | 168960 (26.4) | 165460 (27.5) |
| Yes | 471120 (73.6) | 436073 (72.5) |
| **Total** | **640080 (100.0)** | **601534 (100.0)** |

Table S6: Intimate partner violence during pregnancy (IPVDP) by WHO Regions (with 95% CIs)

| **WHO Regions** | **IPVDP** | **95% CIs** |
| --- | --- | --- |
| African Region (n = 33) | 5.8 | 5.6 - 5.9 |
| Eastern Mediterranean Region (n = 4) | 11.3 | 10.8 - 11.9 |
| European Region (n = 6) | 4.6 | 4.3 - 4.9 |
| Region of the Americas (n = 6) | 9.1 | 8.8 - 9.5 |
| South-East Asia Region (n = 5) | 3.3 | 3.0 - 3.5 |
| Western Pacific Region (n = 3) | 5.1 | 4.7 - 5.6 |

Table S7: Intimate partner violence during pregnancy (IPVDP) by World Bank Income groups (with 95% CIs)

| **World Bank Income groups** | **IPVDP** | **95% CIs** |
| --- | --- | --- |
| Low income (n = 18) | 8.2 | 7.9 - 8.4 |
| Lower-midlle income (n = 28) | 4.9 | 4.8 - 5.1 |
| Upper-midlle income (n = 11) | 7.5 | 7.2 - 7.8 |

Table S8: Intimate partner violence during pregnancy (IPVDP) by countries (latest surveys)

| **No** | **Pays** | **IPVDP** | **WHO Regions** | **World Bank ranking of income** |
| --- | --- | --- | --- | --- |
| 1 | Afghanistan | 15.7 | Eastern Mediterranean Region | Low |
| 2 | Angola | 5.8 | African Region | Lower middle |
| 3 | Armenia | 1.2 | European Region | Upper middle |
| 4 | Azerbaijan | 4.3 | European Region | Upper middle |
| 5 | Benin | 3.8 | African Region | Lower middle |
| 6 | Burkina Faso | 1.3 | African Region | Low |
| 7 | Burundi | 9.8 | African Region | Low |
| 8 | Cambodia | 1.4 | Western Pacific Region | Lower middle |
| 9 | Cameroon | 6.5 | African Region | Lower middle |
| 10 | Chad | 7.5 | African Region | Low |
| 11 | Colombia | 9.7 | Region of the Americas | Upper middle |
| 12 | Comoros | 2.7 | African Region | Lower middle |
| 13 | Côte d'Ivoire | 2.5 | African Region | Lower middle |
| 14 | Democratic Republic of the Congo | 12.5 | African Region | Low |
| 15 | Dominican Republic | 7.4 | Region of the Americas | Upper middle |
| 16 | Egypt | 6.6 | Eastern Mediterranean Region | Lower middle |
| 17 | Ethiopia | 3.7 | African Region | Low |
| 18 | Gabon | 6.8 | African Region | Upper middle |
| 19 | Gambia | 7.4 | African Region | Low |
| 20 | Ghana | 6.8 | African Region | Lower middle |
| 21 | Guatemala | 7.0 | Region of the Americas | Upper middle |
| 22 | Haiti | 5.7 | Region of the Americas | Low |
| 23 | Honduras | 9.2 | Region of the Americas | Lower middle |
| 24 | India | 3.1 | South-East Asia Region | Lower middle |
| 25 | Jordan | 2.7 | Eastern Mediterranean Region | Upper middle |
| 26 | Kenya | 6.5 | African Region | Lower middle |
| 27 | Kyrgyzstan | 7.4 | European Region | Lower middle |
| 28 | Lesotho | 6.3 | African Region | Lower middle |
| 29 | Liberia | 6.5 | African Region | Low |
| 30 | Madagascar | 5.1 | African Region | Low |
| 31 | Malawi | 5.0 | African Region | Low |
| 32 | Maldives | 3.6 | South-East Asia Region | Upper middle |
| 33 | Mali | 6.7 | African Region | Low |
| 34 | Mauritania | 1.2 | African Region | Lower middle |
| 35 | Mozambique | 2.7 | African Region | Low |
| 36 | Myanmar | 3.4 | South-East Asia Region | Lower middle |
| 37 | Namibia | 5.5 | African Region | Upper middle |
| 38 | Nepal | 5.9 | South-East Asia Region | Lower middle |
| 39 | Nigeria | 5.5 | African Region | Lower middle |
| 40 | Pakistan | 7.4 | Eastern Mediterranean Region | Lower middle |
| 41 | Papua New Guinea | 17.6 | Western Pacific Region | Lower middle |
| 42 | Peru | 10.6 | Region of the Americas | Upper middle |
| 43 | Philippines | 3.0 | Western Pacific Region | Lower middle |
| 44 | Moldova | 7.3 | European Region | Lower middle |
| 45 | Rwanda | 5.8 | African Region | Low |
| 46 | Senegal | 3.7 | African Region | Lower middle |
| 47 | Sierra Leone | 6.4 | African Region | Low |
| 48 | South Africa | 1.1 | African Region | Upper middle |
| 49 | Sao Tome and Principe | 6.9 | African Region | Lower middle |
| 50 | Tajikistan | 1.2 | European Region | Low |
| 51 | Timor-Leste | 2.3 | South-East Asia Region | Lower middle |
| 52 | Togo | 7.5 | African Region | Low |
| 53 | Uganda | 10.6 | African Region | Low |
| 54 | Ukraine | 3.7 | European Region | Lower middle |
| 55 | Tanzania | 3.2 | African Region | Lower middle |
| 56 | Zambia | 5.5 | African Region | Lower middle |
| 57 | Zimbabwe | 5.6 | African Region | Lower middle |

Table S9: The AARCs and their 95% CIs in 31 countries (earliest and latest surveys)

| **Countries** | **AARC** | **95% Cis** | |
| --- | --- | --- | --- |
| Burkina Faso | -4,8 | -5,2 | -4,4 |
| Cambodia | -2,8 | -3,3 | -2,3 |
| Cameroon | -3,6 | -4,7 | -2,5 |
| Colombia | 0,0 | -0,5 | 0,5 |
| Côte d'Ivoire | -8,9 | -9,8 | -8,0 |
| Democratic Republic of the Congo | 0,6 | -0,7 | 1,8 |
| Dominican Republic | 1,7 | 0,8 | 2,7 |
| Gabon | -5,6 | -7,1 | -4,2 |
| Gambia | 8,3 | 6,8 | 9,9 |
| Ghana | 2,0 | 1,0 | 3,0 |
| Haiti | -0,8 | -1,9 | 0,2 |
| India | -5,7 | -5,9 | -5,5 |
| Jordan | -4,3 | -5,0 | -3,5 |
| Kenya | -4,3 | -4,9 | -3,6 |
| Malawi | -0,5 | -1,4 | 0,3 |
| Mali | 2,9 | 1,8 | 4,0 |
| Mozambique | -4,1 | -4,9 | -3,3 |
| Nepal | -0,4 | -1,5 | 0,7 |
| Nigeria | 0,8 | 0,1 | 1,5 |
| Pakistan | -6,9 | -8,3 | -5,4 |
| Peru | -1,7 | -2,5 | -1,0 |
| Philippines | -1,4 | -1,9 | -0,9 |
| Rwanda | -3,8 | -5,5 | -2,1 |
| Senegal | 4,5 | 3,1 | 6,0 |
| Sierra Leone | -4,4 | -5,6 | -3,3 |
| Tajikistan | -24,9 | -25,6 | -24,2 |
| Timor-Leste | -6,9 | -7,7 | -6,0 |
| Uganda | -3,9 | -5,0 | -2,7 |
| United Republic of Tanzania | -8,4 | -9,4 | -7,3 |
| Zambia | -5,0 | -5,8 | -4,1 |
| Zimbabwe | -4,0 | -5,0 | -3,1 |

AARC=average annual rate of change.

Notes: A negative AARC value indicates a reduction in the prevalence of IPVDP,

and a positive value, an increase.

Fig. S1: Variations of intimate partner violence during pregnancy (IPVDP) against women aged 15 to 49 years in 31 countries, using the earliest and the latest DHSs


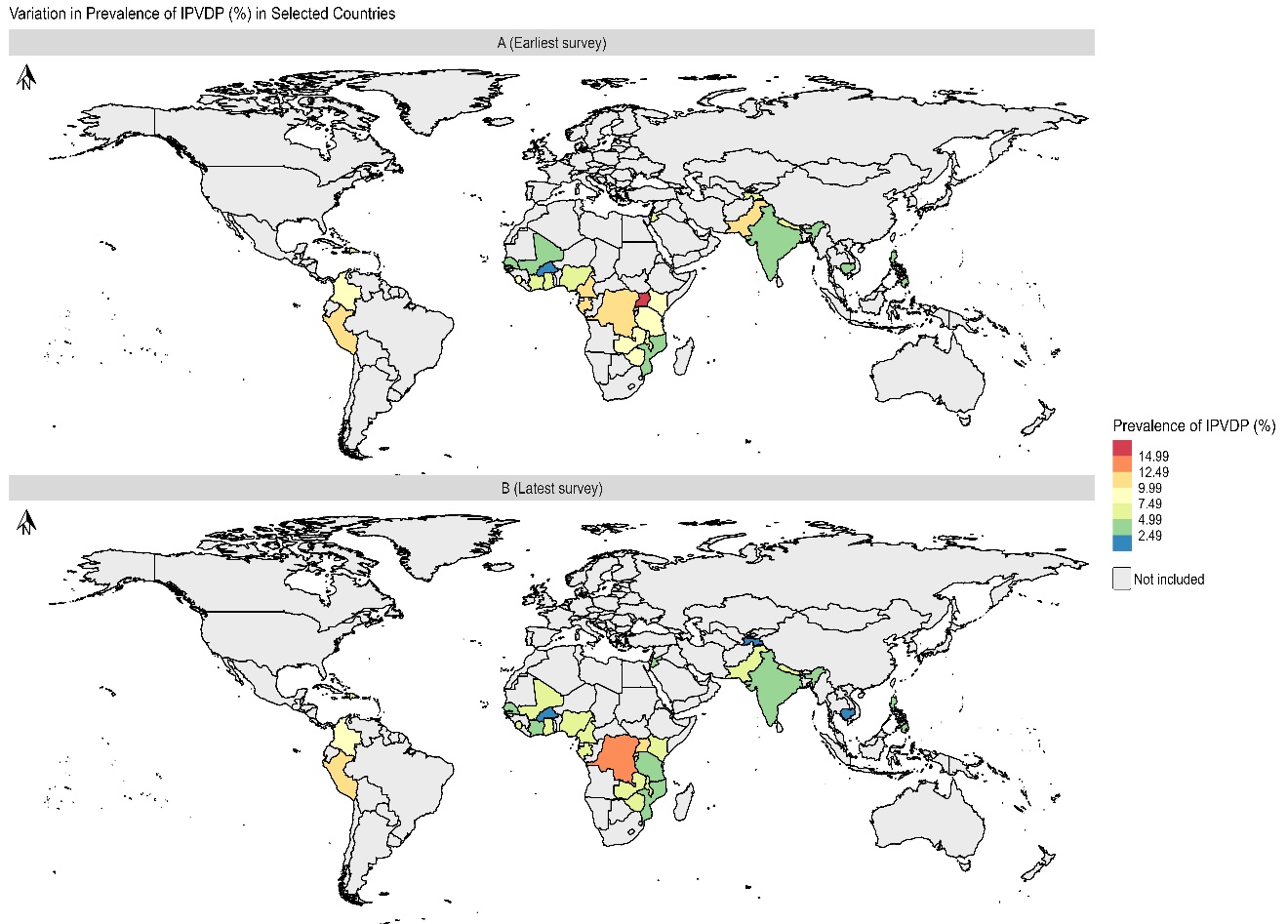


Notes: Countries highlighted in grey are those for which data are unavailable.

Table S10: Intimate partner violence during pregnancy (IPVDP) prevalence by selected socio-demographic characteristics (using latest DHS)

| **Socio-demographic  characteristics** | **Women aged 15-49 years experienced IPVDP** | | **χ² (*p*-value)** |
| --- | --- | --- | --- |
|  | Yes [N (%)] | No [N (%)] |  |
| **Age** |  |  | < 0.001 |
| 15-19 | 1150 (7.1) | 15095 (92.9) |  |
| 20-24 | 3348 (6.2) | 50540 (93.8) |  |
| 25-29 | 4397 (6.4) | 63986 (93.6) |  |
| 30-34 | 4325 (6.5) | 62033 (93.5) |  |
| 35-39 | 3849 (6.2) | 58618 (93.8) |  |
| 40-44 | 2946 (5.7) | 49005 (94.3) |  |
| 45-49 | 2983 (6.4) | 43452 (93.6) |  |
| **Place of residence** |  |  | < 0.001 |
| Urban | 9590 (6.1) | 147522 (93.9) |  |
| Rural | 13427 (6.4) | 195206 (93.6) |  |
| **Women's education level** |  |  | < 0.001 |
| Primary and below | 13975 (7.5) | 172770 (92.5) |  |
| Secondary | 7342 (5.5) | 127136 (94.5) |  |
| Higher | 1680 (3.8) | 42820 (96.2) |  |
| **Wealth Index** |  |  | < 0.001 |
| Poor | 10368 (7.2) | 133253 (92.8) |  |
| Middle | 5249 (7.0) | 69985 (93.0) |  |
| Rich | 7380 (5.0) | 139490 (95.0) |  |
| **Number of children** |  |  | < 0.001 |
| Zero | 528 (4.0) | 12653 (96.0) |  |
| 1-2 | 8599 (5.1) | 158805 (94.9) |  |
| 3-5 | 9330 (6.9) | 126386 (93.1) |  |
| More than 5 | 4540 (9.2) | 44883 (90.8) |  |
| **Age at first union** |  |  | < 0.001 |
| Under 18 | 10018 (7.5) | 123285 (92.5) |  |
| 18 and above | 12979 (5.6) | 219443 (94.4) |  |
| **Currently working** |  |  | < 0.001 |
| No | 8211 (5.4) | 144389 (94.6) |  |
| Yes | 14787 (6.9) | 198339 (93.1) |  |
| **Partner’s controlling behaviour** |  |  | < 0.001 |
| No controlling behaviour | 3653 (2.4) | 145651 (97.6) |  |
| Has controlling behaviour | 19344 (8.9) | 197077 (91.1) |  |
| **Participation in household decision-making** |  |  | < 0.001 |
| No | 7985 (8.1) | 90446 (91.9) |  |
| Yes | 15012 (5.6) | 252282 (94.4) |  |
| **Total** | **22997 (6.3)** | **342728 (93.7)** |  |
